# Supplementary material for: Circulating Levels of Epirubicin Cause Endothelial Senescence While Compromising Metabolic Activity and Vascular Function
Source: Front Cell Dev Biol. 2020 Aug 19;8:799. doi: 10.3389/fcell.2020.00799 (PMC7466755; doi:10.3389/fcell.2020.00799)
Supplement: Supplementary file 1 [file Image_1.pdf]

Supplementary Figure S1

Effects of epirubicin exposure on cell density and morphology

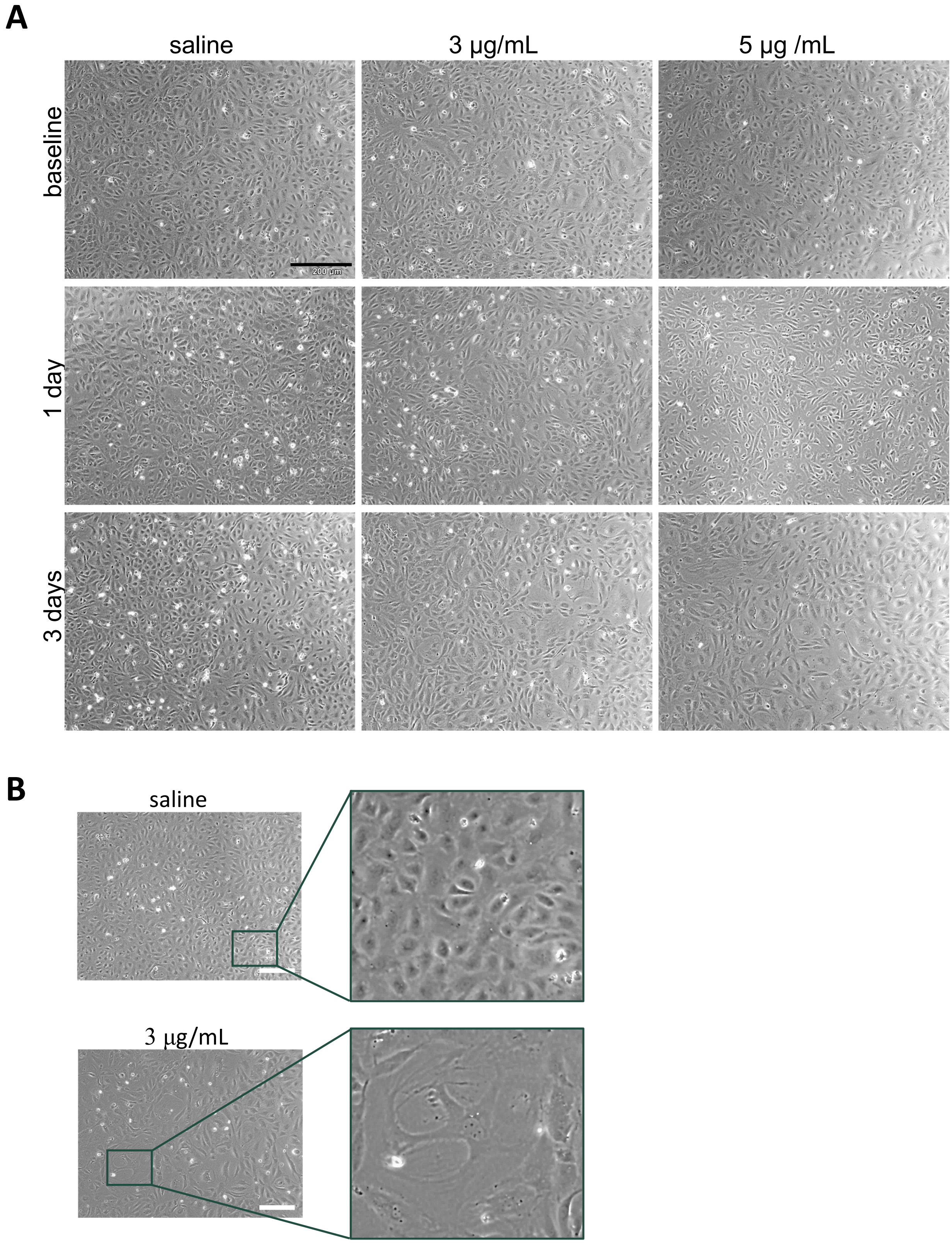

(A) Confluency images of various time points throughout the experiment. Changes in confluency were observed using an EVOS phase contrast microscope (10x magnification). (B) Representative images and enlarged insets to illustrate senescent-like morphology of endothelial cells following exposure to Epirubicin. Images were taken at 10 x magnification 7 d following initial treatment with saline or 5 μg/mL Epirubicin. Scale bars represent 200 μm.

Supplementary Figure S2

Viability control prior to functional assays (A) and effects of epirubicin on EC junctions (B)

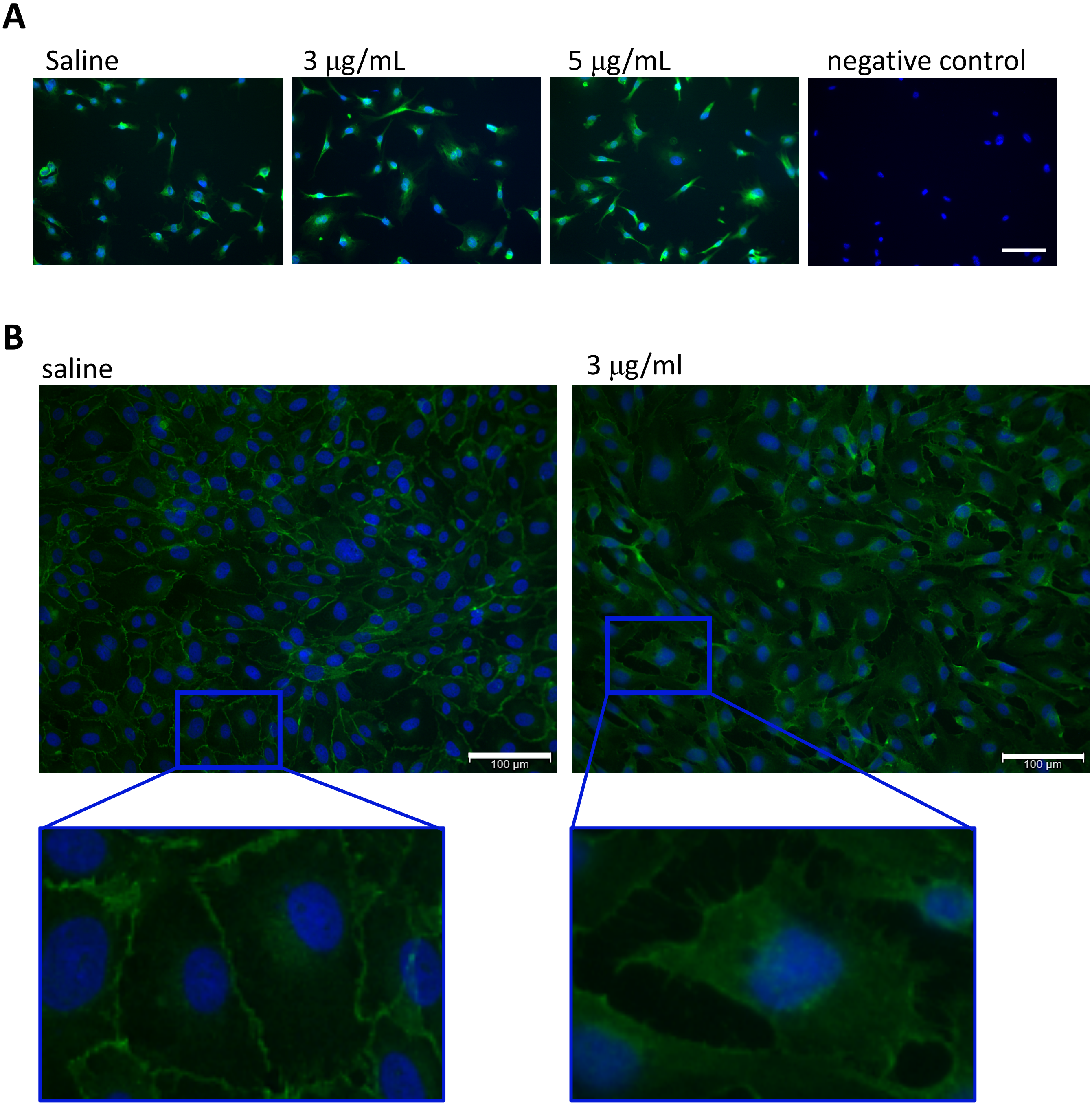

(A) Cells used for functional assays (main Figure 3) were stained with Calcein AM at the last time point to ensure viability of experimental cell population throughout the assays. DAPI is shown in blue, and calcein in green. (B) VE-Cadherin staining (green) of hUVEC 1 d after treatment with 3 μg/mL Epirubicin of respective saline control. Counterstained with DAPI (blue). scale bars on A and B = 100 μm.

Supplementary Figure S3

Epirubicin affects transcriptional activity in endothelial cells

(A)

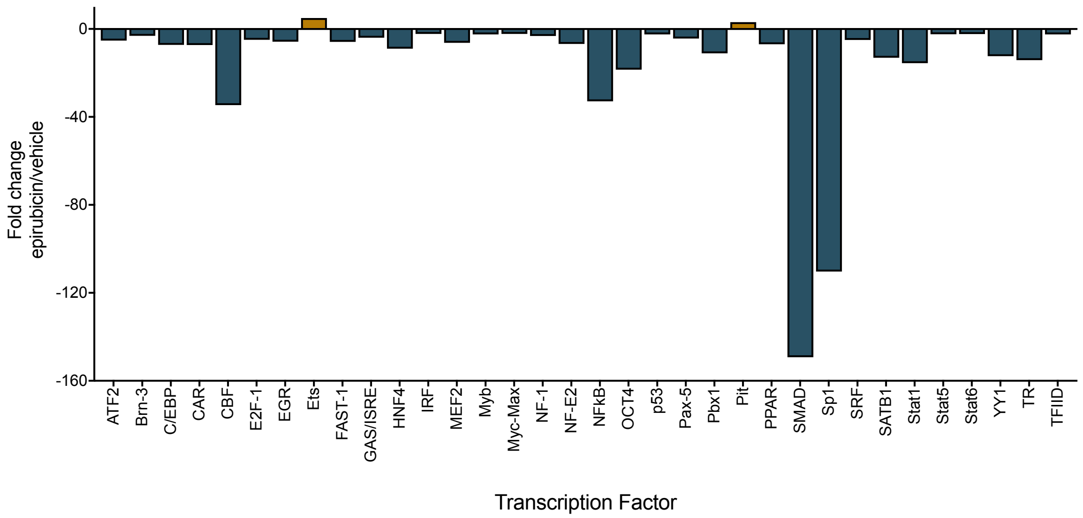

(B)

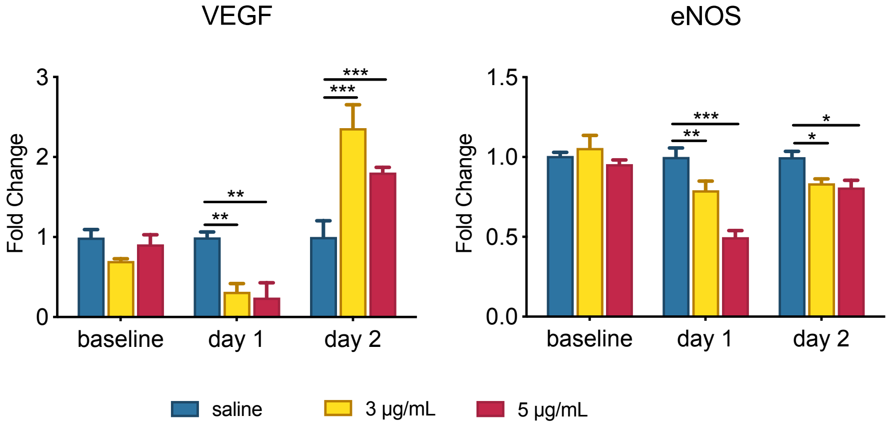

(A) Transcription factor activation screen (TF Activation Profiling Plate Array I - Signosis) of hUVEC 1 d after treatment (30 min) with either vehicle or 3 µg/mL Epirubicin was quanfified using an Omega Plate reader (BMG Labtech). As per manufacturer's instructions, only transcription factors outside ± 10% of the blank sample and with a fold change > 2 compared to control samples are considered significant, and as such only TF obeying that criteria are displayed; (B) Transcript levels for key endothelial cell function factors, VEGF and eNOS, were quantified at baseline and after 1 d and 2 d after exposure to Epirubicin; data represents average fold change ± SE; Statistical significance was assessed by Two-Way ANOVA with Bonferroni Correction, and *p<0.05, **p<0.01, ***p<0.001, n=2.
